# Biological Properties of Solid Free Form Designed Ceramic Scaffolds with BMP-2: *In Vitro* and *In Vivo* Evaluation

**DOI:** 10.1371/journal.pone.0034117

**Published:** 2012-03-28

**Authors:** Ander Abarrategi, Carolina Moreno-Vicente, Francisco Javier Martínez-Vázquez, Ana Civantos, Viviana Ramos, José Vicente Sanz-Casado, Ramón Martínez-Corriá, Fidel Hugo Perera, Francisca Mulero, Pedro Miranda, José Luís López-Lacomba

**Affiliations:** 1 Instituto de Estudios Biofuncionales, Universidad Complutense, Madrid, Spain; 2 Centro Nacional de Microbiología, Instituto de Salud Carlos III, Majadahonda, Madrid, Spain; 3 Departamento de Ingeniería Mecánica, Energética y de los Materiales, Universidad de Extremadura, Badajoz, Extremadura, Spain; 4 Departamento de I+D, Noricum S.L., San Sebastián de los Reyes, Madrid, Spain; 5 Facultad de Medicina, Universidad Complutense, Madrid, Spain; 6 Molecular Imaging Core Unit, Centro Nacional de Investigaciones Oncológicas, Madrid, Spain; Faculty of Medicine University of Leipzig, Germany

## Abstract

Porous ceramic scaffolds are widely studied in the tissue engineering field due to their potential in medical applications as bone substitutes or as bone-filling materials. Solid free form (SFF) fabrication methods allow fabrication of ceramic scaffolds with fully controlled pore architecture, which opens new perspectives in bone tissue regeneration materials. However, little experimentation has been performed about real biological properties and possible applications of SFF designed 3D ceramic scaffolds. Thus, here the biological properties of a specific SFF scaffold are evaluated first, both *in vitro* and *in vivo*, and later scaffolds are also implanted in pig maxillary defect, which is a model for a possible application in maxillofacial surgery. *In vitro* results show good biocompatibility of the scaffolds, promoting cell ingrowth. *In vivo* results indicate that material on its own conducts surrounding tissue and allow cell ingrowth, thanks to the designed pore size. Additional osteoinductive properties were obtained with BMP-2, which was loaded on scaffolds, and optimal bone formation was observed in pig implantation model. Collectively, data show that SFF scaffolds have real application possibilities for bone tissue engineering purposes, with the main advantage of being fully customizable 3D structures.

## Introduction

Porous bioceramics are widely used in medical applications as bone substitutes or as bone-filling materials [1]–[4]. These porous scaffolds are used to provide structural support and also to serve as a template for cell colonization and extracellular matrix formation [5]. Both degradable and non-degradable ceramics are used to fabricate scaffolds and also multiple methods have been used to create the porous structure [6]–[8].

However, most conventional scaffold fabrication methods do not allow the fabrication of structures with customized and complex external shapes or internal pore architectures. Solid free form (SFF) fabrication techniques - three-dimensional printing, stereolithography, fused deposition modeling, robocasting, phase-change jet printing, etc. - constitute an excellent alternative to produce well-defined 3D structures [9]–[13]. These SFF technologies involve building 3-D objects from a computer-aided design (CAD) model using layered manufacturing strategies. An additional advantage of SFF scaffolds is that potentially they could be specifically designed for specific bone defects, for example, taking as model an x-ray tomography image.

Robocasting, also known as direct-write assembly or microrobotic deposition, is a SFF method that consists of the robotic deposition of water-based colloidal suspensions (inks), with a high solid-load and a minimal organic content (∼1 wt.%), capable of fully supporting their own weight during assembly [14], [15]. Deposition is usually made within an oil bath to prevent non uniform drying during assembly. Thus, a 3D network of semisolid ink rods is created layer-by-layer without the need for a sacrificial support material or mould.

Recent work has been directed towards developing ceramic robocast structures [16]–[18] with the aim of combining the excellent biological properties of the bioceramics with those provided by a fully controlled, reproducible and customizable architecture. Extensive material characterization has been performed in order to elucidate the mechanical properties of these scaffolds [16], [17], [19], [20]. However, as yet, there is no biological information available to confirm the expected applicability of free form designed, architecturally well-defined ceramics.

*In vitro* biological properties [21]–[23] and prospective *in vivo* assays [12], [24]–[28] are already performed with other SFF designed scaffolds, which were made of different materials as polymers, composites and ceramics. Thus, the purpose of this work is biological and it is related firstly to testing the biocompatibility and bioactivity of these scaffolds, and later to assess the potential of this approach to become clinically viable. In addition, the benefits of incorporate an osteoinductive factor in the robocast scaffolds are also explored in this work. BMP-2 is a well-known osteoinductive growth factor that combined with porous ceramics improves the osteointegration [29]–[32].

## Results and Discussion

### 1. Scaffold presentation and morphology

**Figure 1:**
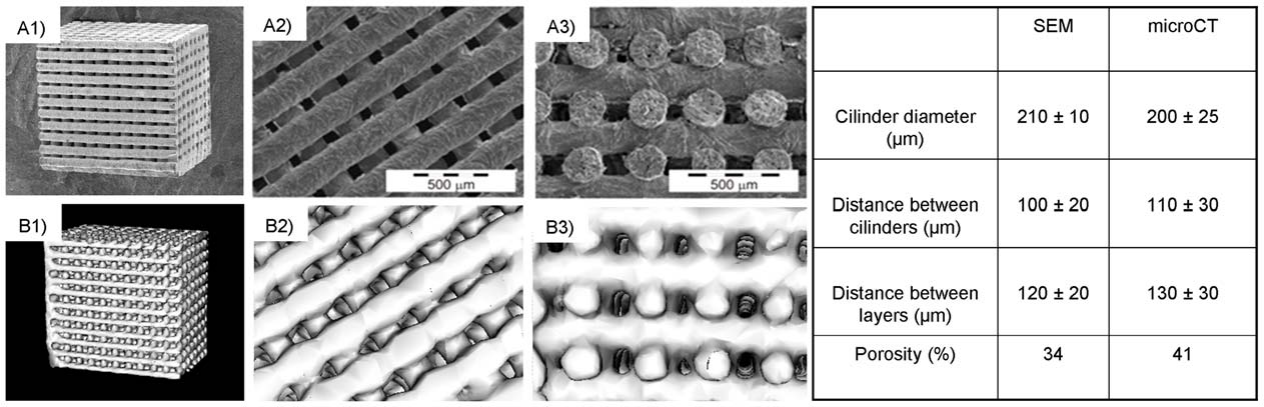
SFF designed Scaffold. A) SEM micrographs of scaffold samples. A1) Entire scaffolds; A2) top plane view; A3) cross-section view. B) μCT images of scaffold samples. B1) Entire scaffolds; B2) top plane view; B3) cross-section view. Table shows measured structural data.

Material fabrication process and physical properties have been previously described [16], [17], [19], [20]. It is well known the importance of some scaffold properties in tissue conduction processes [33], [34]. Features like scaffold geometry and surface properties are biologically relevant, since they have a strong influence in cell adhesion and proliferation processes [35], [36]. For testing assays square scaffolds were designed. Structural data are summarized on figure 1 (see table) and macrostructure is also shown in Figure 1, imaged by SEM (fig. 1A) and μCT (fig. 1B). Views of the entire scaffolds (fig. 1A1, 1B1), top surface (fig. 1A2, 1B2) and transversal section (Fig. 1A3, 1B3) are presented. Images correspond to different samples.

### 2. *In vitro* testing

Cellular assays were performed in order to assess cell adhesion, viability, proliferation and migration on mentioned scaffolds. For these assays C2C12 pre-myoblastic cell line was selected. As adherent cell line, it allows testing cell adhesion and colonization of scaffold surfaces. In addition, C2C12 cell line has the ability to evolve to an osteoblastic phenotype upon the addition of BMP-2 [37]–[41]. BMP-2 modifies multiple cellular processes in C2C12 cells, as cell adhesion, proliferation and migration, thereby inducing osteodifferentiation and processes implicated in new bone tissue vascularization and progression [40]–[42].

**Figure 2:**
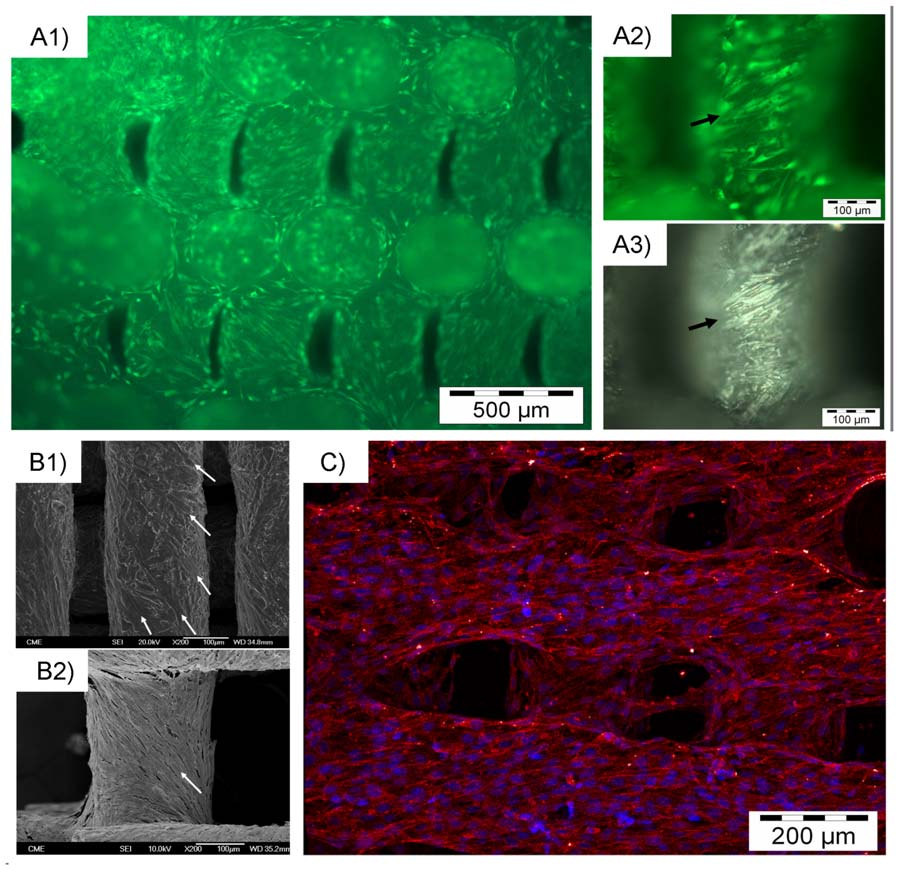
Cell adhesion studies. A) Calcein cell viability assay. A1) Fluorescence image of a cell-seeded scaffold after 3 days. Green cells are viable cells. A2) A detailed image showing green viable cells adhered to the material. A3) Light image of the same sample area. Arrows in A2 and A3 mark similarities between surface morphology and cellular pattern. B) SEM images. B1) Image of a control, unseeded scaffold. Arrows indicate extrusion marks in the surface of the ceramic rods. B2) A cell seeded scaffold after 7 days. Note that cells follow the pattern of the surface morphology indicated in B1 image. C) Confocal image of a cell covered scaffold surface at seventh culture day. (Red, actin cytoskeleton; Blue, Nucleous).

Thus, firstly cell adhesion and spreading was checked. Figure 2 shows micrographs of different methods used to visualize cells on scaffolds surface. The green fluorescence observed on Fig. 2A1 and 2A2 corresponds to calcein vital staining, and shows viable cells adhered on the scaffold surface. A comparison of fluorescent (Fig. 2A2) and white light (Fig. 2A3) images shows that adhered cells tend to follow the material surface pattern. Further evidence of this is shown in the SEM images (Fig. 2B); On the unseeded scaffold (Fig. 2B1) a pattern of extrusion marks is evident on the material's rod surfaces, while the image of a fully cell-covered scaffold (Fig. 2B2) shows the tendency of cells to follow the scaffold's irregular, grooved surface. Also cell nucleous and actin cytoskeleton were stained and visualized in a confocal microscope (Fig. 2C: blue, nucleous; red, actin cytoskeleton). Images show again a fully cell-covered scaffold surface, assessing the ability of the scaffolds to promote cell adhesion and growth.

**Figure 3:**
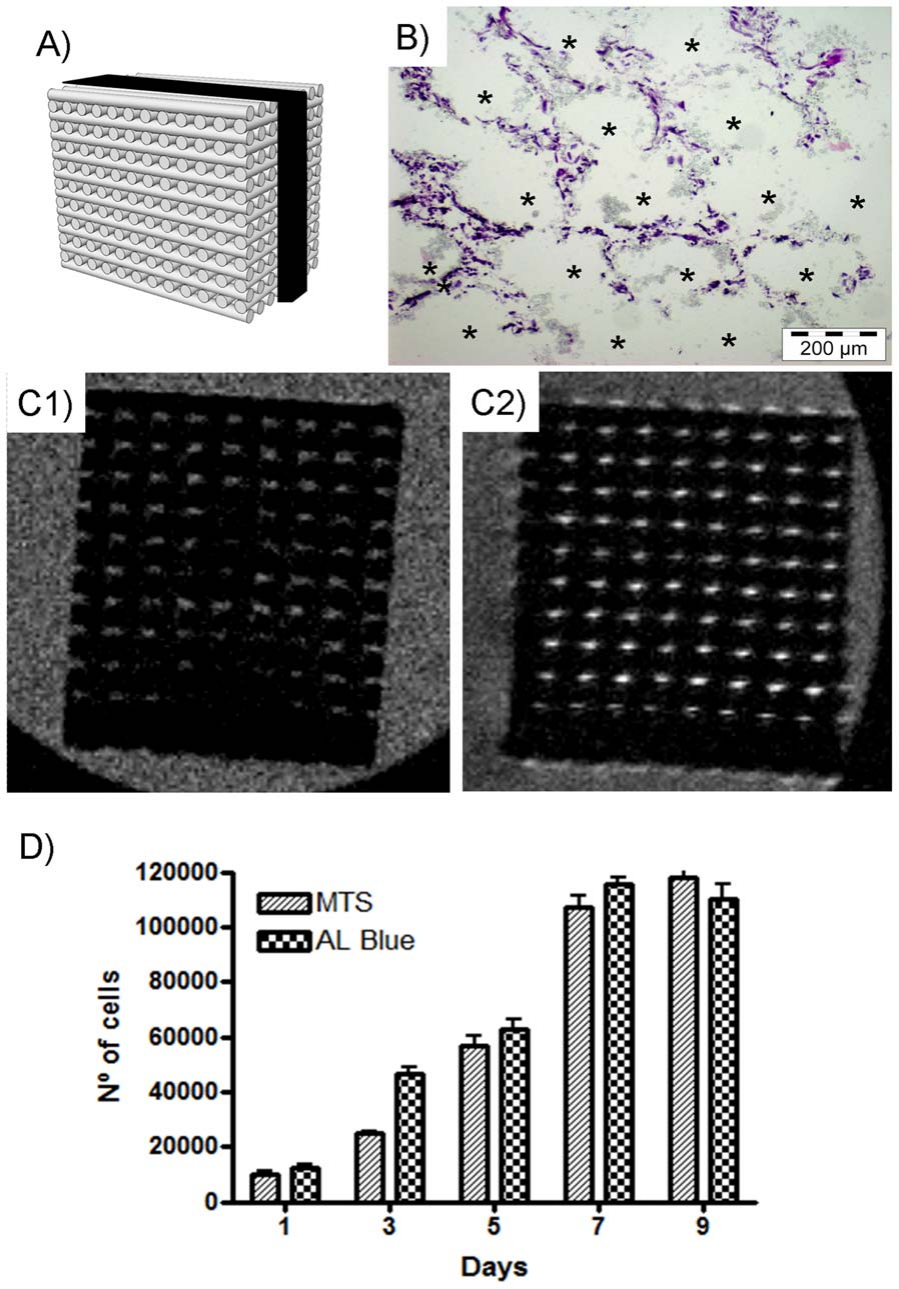
Inner-cells studies and quantification assays. A) Scheme of cell-location studies. Black area correspond to studied area. B) Histology inner the robocasting structure. Asterisks show the location of ceramic rods, which are empty spaces due to performed sample decalcification. Note C2C12 cells located in entire scaffold structure. C) Non-destructive MRI images obtained inner scaffold structure. C1) Non cell-seeded scaffold C2) Cell seeded scaffold. Note brightness in most porous structure attributable to cell presence. D) Quantitative assays for cell proliferation measurement. Graphic represents the time course plot obtained in both MTS and Alamar Blue assays.

Figure 2 demonstrates good cell adhesion and spreading on the material surface, and suggests an influence of the scaffold surface patterning on the cell distribution and shape. However, a critical feature in porous scaffolds is the capability for seeded cells to migrate and grow on the inner surfaces. Figure 2 shows partially cells penetrating inside scaffolds but microscopic techniques are not enough to assess it. Thus, histological and MRI assays were performed in order to observe cells deep inside structure. Figure 3A shows in black the area in which these assays were performed. Histology is already used for other 3D material testing [21]. Figure 3B corresponds to a histological slice obtained in the center of the specimen and it shows cells inner scaffold's structure. MRI technique was also used to see/check live cells inner scaffold structure. Figure 3C1 shows a non cell-seeded material while figure 3C2 shows a cell seeded scaffold. There bright areas in the porous structure correspond to live cells. Additionally, 3D MRI studies were performed and cells were located in entire scaffold structure (see Movie S1).

All previous results show cells in the surface or inside material structure, but cell proliferation should be assessed in order to consider a material biocompatible. The proliferation of cells was quantified using two complementary methods, which are MTS and alamar blue tests. The time course plot of these assays (Fig. 3D) shows the cell proliferation behavior in the scaffold. As it can be observed, initially ∼10000 cells adhered to the scaffold, while subsequent measurements show good cell proliferation on the material. Doubling time of cells on scaffolds was estimated in 32,1 hours, which is similar to the doubling time of C2C12 cells in control plastic cell culture surface (29,7 hours).

In bone tissue engineering osteoinductive factors are usually included to scaffolds, via factor-adsorption onto the surface or via factor-entrapping in a carrier material [43]–[46]. BMP-2 is a well known osteoinductive factor [30], [31], [41], [47]–[49] and this property is desirable if *in vivo* bone formation is desired. Here we selected and tested two delivery methods which have been previously reported, both *in vitro* and *in vivo*, with BMP-2 and other scaffolds: 1- surface adsorption [32], [50], which is later *in vivo* assayed in models 3.1 and 3.2, and 2- entrapping in a coating material (Chitosan) [40]–[42] which is later shown in *in vivo* model 3.3. These methods were selected because there are several paper focused on BMP2 adsorption on ceramic scaffolds and subsequent delivery and also because we previously reported BMP-2 delivery from chitosan coating [42].

**Figure 4:**
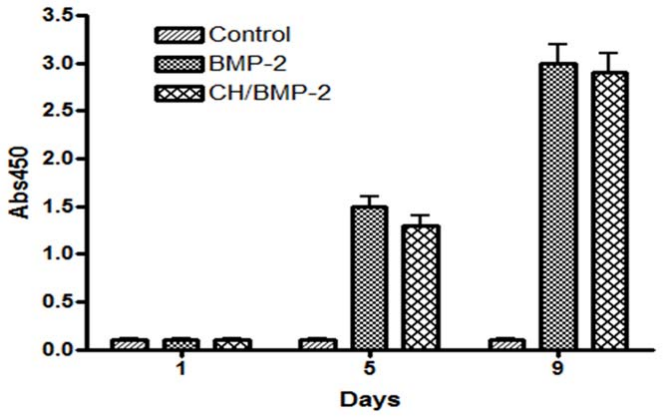
ALP activity measurements. Time course plot of ALP activity in control scaffolds (Control), BMP-2 adsorbed scaffolds (BMP-2) and Chitosan/BMP-2 coated scaffolds (CH/BMP-2).

The activity of this growth factor on the scaffold surface was *in vitro* assessed by measuring the ALP activity (an osteoblastic differentiation specific marker) of scaffold-adhered cells after the first, fifth and ninth culture day. Plot in Fig. 4C shows that ALP activity appears at fifth culture day and grows at ninth when BMP-2 is present on scaffold. These data indicate that BMP-2 on scaffolds is active and induces *in vitro* bone differentiation. In contrast, cells seeded on the control scaffolds do not present ALP activity in any case.

All together, *in vitro* results show that these scaffolds are adequate for cell attachment, proliferation and colonization of entire structure, thereby confirming that these SFF-designed materials have appropriate biological properties, at least *in vitro*.

### 3. *In vivo* assays

Bone tissue regeneration is the main applicability of ceramic scaffolds. However, in scaffold field, a gap exists between research and clinical translation [51]–[55]. Thus, focused on a translational approach, we decided to perform an exhaustive *in vivo* testing, both in small and large animal models, both in ectopic and orthotopic models, and also both in delayed bone healing model and critical size bone defects, in order to verify material properties. Thus, assays were designed in order to firstly check the conductive effect of the control scaffolds and later confirm osteoinductive property of scaffold/BMP-2 samples.

#### 3.1. Rabbit: Muscle Implantation

**Figure 5:**
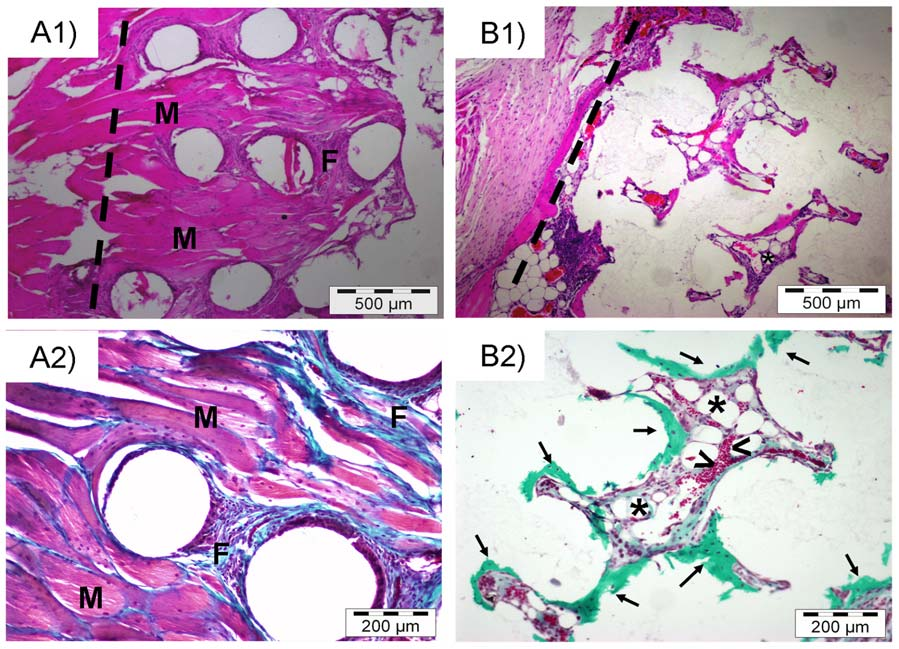
Implantation in rabbit muscle. A) control scaffolds, B) BMP-2 adsorbed scaffolds: 1) Hematoxilin/Eosin stainings, 2) Massons tricrome stainings. Dotted line marks scaffold limits. Empty round spaces correspond to the ceramic material. A1, A2) Fibrous tissue (F) and muscle tissue (M) are present in the structure of the control scaffolds. B1, B2) Newly formed bone (green tissue: arrows, ↑), fatty tissue with vessels (asterisks, *) and red blood cells (arrow heads, >) are observed in BMP-2 adsorbed scaffolds.

Initially biological behavior of scaffolds was tested by implantation of samples in rabbit dorsal muscle tissue. This ectopic model allows the testing of both conductive effect of scaffold's structure and osteoinductive effect of incorporated BMP-2. Fig. 5 shows histological appearance of samples implanted during 3 weeks. Control scaffolds (Figures 5A) show a matrix filled of muscle tissue which comes from the surrounding to inside material. Also fibrous tissue can be observed. On the other hand, scaffolds with BMP-2 exhibit a cell invaded matrix (Fig. 5B), but the appearance is completely different. In these cases, fatty tissue with vessels containing red blood cells can be observed between large areas of newly formed bone (Fig. 5B2, green tissue). It indicates an advanced bone formation inside the material with generation of fatty marrow spaces.

These results show that the macropore structure allows surrounding tissue colonize inner space of robocast scaffold. However, the influence of the BMP-2 in the newly formed tissue was remarkable (Fig. 5): muscle tissue and fibrous tissue were observed inside the control scaffolds, while ectopic bone tissue was formed inside BMP-2 activated scaffolds. This bone formation associated with marrow suggests that BMP-2 and scaffold structure allow a direct bone-inducing effect [43], [44].

#### 3.2. Rabbit: Bone Implantation

**Figure 6:**
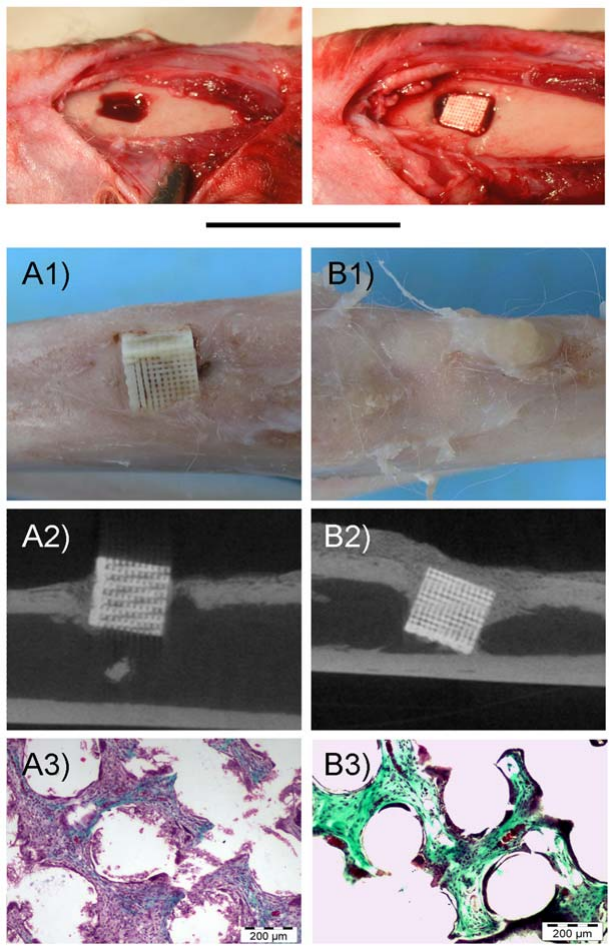
Implantation in rabbit bone. Up, images of the surgical procedure. A) control scaffolds, B) BMP-2 adsorbed scaffolds. 1) Gross appearance of harvested samples, 2) Representative μCT slides, 3) Representative histological images (Massons tricrome stainings). Note in B1 and B2 bone formation outside structure, which covered all the scaffold. See in A3 and B3 the tissue formed inside structure. Fibrous tissue is observed as purple-blue while bone is green.

These findings were confirmed in a second *in vivo* model in an orthotopic location. In this case, bone defects were created in rabbit tibia and samples were implanted (fig. 6). Figure 6 shows surgery procedure and sample appearance 3 weeks after surgery. Control samples were stable in the bone tissue and look integrated, while BMP-2 samples show high amount of bone formation onto and around the implanted material. The representative μCT slides in Figure 6 show no bone around the control scaffolds (fig. 6A2) while newly formed bone is clearly observable surrounding the BMP-2 carrier scaffolds (fig. 6B2). Furthermore, the histological study shows that fibrous tissue was formed deep inside control implanted scaffolds (Fig. 6A3) while in the BMP-2 carrier scaffolds new bone tissue was the observed one within the matrix (Fig. 6B3). In this case, like in muscle implantation model, fatty tissue and red blood cells are also but barely appreciable, being bone most of the formed tissue.

These results in rabbit models indicate that material on its own conducts surrounding tissue and allow cell ingrowth, thanks to the scaffold structure. In addition, scaffolds with BMP-2 show also osteoinductive properties.

#### 3.3. Pig Maxillary tissue: Clinically relevant animal model

In order to extend on the applicability of these structurally customizable scaffolds, a specific design was fabricated and pig, which is physiologically closer to human being, was used as animal model. In odontology lack of enough bone tissue volume to work is a usual clinical problem. Thus, critical size superior maxillary defects was used as model of poor bone tissue volume area. Robocast/BMP-2 scaffold was tested as bone augmentation agent and samples were specifically designed in order to allow their immobilization by two screws. With comparative purposes, clinically available conventional porous ceramic blocks (Bio-Oss®) with irregular internal pore structure were also implanted.

**Figure 7:**
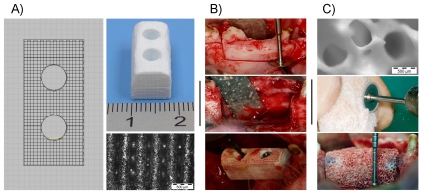
Implantation in pig maxillary defects: materials and surgery. A) Robocast ceramic design, macroscopical appearance of scaffold and microscopical detail. B) Images of surgery procedure, implantation of a Robocast sample and fixation of it with screws. C) Microscopical image of a Bio-Oss® sample, detail of Bio-Oss® sample preparation procedure and Bio-Oss® implanted and fixated with surgical glue (see blue glue between sample and surrounding bone).

Figure 7A shows specifically designed material and its structural properties. Surgery is shown in fig. 7B where defect, Robocast material (which fits to performed defect) and screw implantation could be observed. Fig. 7C also shows implanted Bio-Oss® blocks, which were mechanically polished in order to fit to the defect area and which required surgical glue in order to fix them to defects.

**Figure 8:**
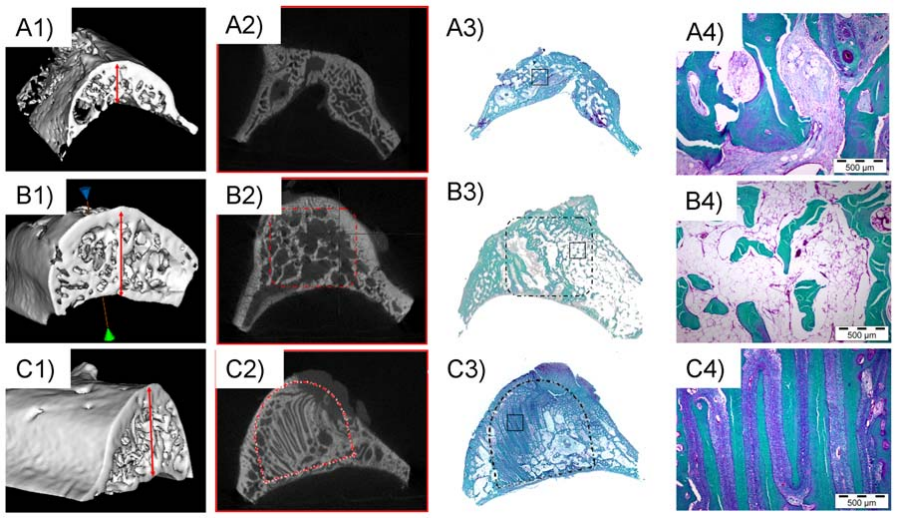
Implantation in pig maxillary defects: Results. A) Control empty sample. B) Bio-Oss/BMP-2 sample. C) Robocast/BMP-2 sample. 1) 3D reconstruction of μCT studies. Red arrows indicate bone height. 2) Representative μCT slices. Dotted lines indicate scaffold location. 3) Macroscopic appearance of histological samples. Dotted lines indicate scaffold location. Squares indicate the area observed in provided histological details (4).

Three months after surgery samples were harvested and fig. 8 summarizes obtained results. BMP-2 loaded samples showed high amount of newly formed bone in the defect area, compared to empty control and control ceramic scaffolds. Fig. 8 shows it in images which correspond to performed microCT and histological studies. Differences in bone volume are appreciable in microCT images, while histologies show differences in the newly formed bone structure due to implanted materials. Bone follows material internal structure and, being robocast structure regular, it could be observed in histological samples (see fig. 8C4, which correspond to the centre of a sample, in the area between screws).

**Figure 9:**
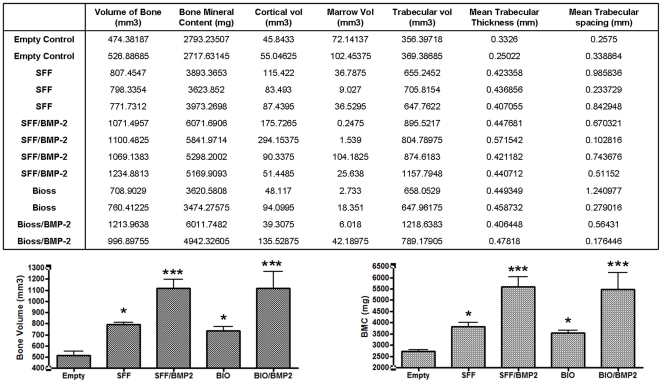
Implantation in pig maxillary defects: Data. Table summarized data obtained from μCT studies (SFF, Robocast scaffolds; BIO, Bio-Oss). Graphic represent “Bone Volume” and “Bone Mineral Content” (BMC). Data in graphics are provided in mean and standard deviation. Significative differences stand for: * (p≤0.05), ** (p≤0.01), *** (p≤0.001).

The differences shown in figure 8 were measured in 13 from all 16 samples, and obtained microCT data are summarizes in figure 9. Although some treatments were tested only in duplicate, data show statistical differences in bone formation between empty and all other treatments (p≤0.05 for control and ceramic scaffolds, p≤0.001 for SFF/BMP-2 and Bio-Oss®/BMP-2 scaffolds). No differences were observed between control Robocast and Bio-Oss® scaffolds and neither between BMP-2 carrier materials. Thus, it could be concluded that tested Robocast scaffolds have similar osteoconductive properties to an already clinically available material (Bio-Oss®) and show similar osteoinduction due to BMP-2 activation. However, SFF ceramic scaffolds could be specifically designed depending on defect and additionally also facilitate surgery procedure.

### 4. Conclusions

SFF designed ceramic scaffolds have the great advantage that external shape and macroporosity architecture can be precisely controlled by the spacing of the deposited ink rods. This work shows the biocompatible and conductive properties of custom-designed ceramic scaffolds. Osteoinductive properties have been added via incorporation of BMP-2 in structure. A scaffold was specifically designed taking into account a clinical problem and results show the potential applicability of this scaffolding method. Future work would be directed to study specifically designed structures and external shapes for specific applications in odontology, traumatology and bone cancer surgery.

## Materials and Methods

### 1. Ethics Statement

All animal handling and experimental procedures were approved by the Animal Care and Usage Committee of Universidad Complutense and Hospital Clínico San Carlos, according to the guidelines for ethical care of experimental animals of the European Community.

### 2. Scaffold fabrication

The preparation of the HA and β-TCP scaffolds can be found elsewhere [16], [18]. Briefly, commercially available ceramic powders (Fluka, Buchs, Switzerland), with an average particle size of 1.8±0.8 µm, were used to prepare inks for robocasting. The inks were used to build scaffolds layer-by-layer with a total of 44, using Robocad 3.0 (3D Inks, Stillwater, OK, USA) for computer design, in which the in-plane line spacing (from center to center) of the rods was set to 400 µm and the layer spacing at 225 µm. A three-axis robotic arm moved the injection syringe while pressing the ceramic ink through conical deposition nozzles of 250 µm diameter, immersed in an oil bath, to create the self-supporting 3-D networks. The resulting samples were dried in air at room temperature for 24 h and then at 400°C for 1 h to evaporate organics, followed by a sintering treatment at 1300°C for 2 h. Then, the scaffolds were cut to 3×3×3 mm pieces. Previously to the *in vitro* and *in vivo* experiments pieces were heat sterilized at 300°C.

### 3. BMP-2 incorporation on scaffolds

*Escherichia-coli*-produced recombinant human BMP-2 (rhBMP-2) was generously supplied by Noricum S.L. (Spain). BMP-2 was adsorbed onto robocasting surface (500 µg/piece) as described in previous works for other porous ceramic scaffolds [32], [50]. Briefly, each piece was immersed in 400 µL of a BMP-2 solution (1.25 mg/mL, 50 mM acetic acid). Then, the samples were incubated in a sterile laminar flow hood at room temperature. The absorbance at 280 mm was measured in the remaining adsorption media, in order to ensure that all the BMP-2 was absorbed on the scaffold. For pig implantation model, BMP-2 was incorporated to scaffolds via chitosan/BMP-2 coating (1 mg of BMP-2/piece) as it has been described elsewhere [40]–[42].

### 4. Microcomputed tomography (μ-CT)

A μCT system (eXplore Vista, GE) was used to perform non-destructive imaging and to quantify the 3D microarchitectural morphology of each sample. Samples were imaged with an X-ray tube voltage of 50 kV and a current of 200 µA. The scanning angular rotation was 180°, the angular increment 0.40°, and the voxel resolution 50 µm. Data sets were reconstructed and segmented into binary images (8-bit BMP images) for the subsequent image processing, measurements and 3D surface reconstructions using MicroView ABA 2.2 software (GE Healthcare).

### 5. Scanning electron microscopy (SEM)

The samples were gold-sputtered (Pelco 91000 sputter coater). A scanning electron microscope (SEM, JSM-6330F Jeol, Japan) was used to characterize the surface topography of samples.

### 6. Cell culture

C2C12 mouse muscle myoblastic cell line was used (CRL 1772, ATTC, USA). Cells were cultured in DMEM high in glucose (31966-021,GIBCO, UK), containing 10% Fetal Bovine Serum (10500-064, GIBCO, UK) plus antibiotics (100 U/ml penicillin and 100 µg/ml streptomycin sulphate) (GIBCO,UK). Culture conditions were 37°C in a humidified 5% CO_2_ atmosphere. Cell passaging was always performed at 80% of confluence.

### 7. General set up for all cellular assays

Each sterile scaffold was placed into a well of 48-well plates, and trypsinized cells were seeded (50000 cells/sample). Afterwards, 400 µl of pre-warmed complete culture medium was added on each well. Before any measurement, each scaffold was transferred to a new culture well to avoid contaminations into the results by cells adhered onto the plastic surface of the well. All assays were done at least in triplicate.

#### 7.1. Cellular viability assay

Cellular viability was tested after three days of culture with the calcein AM assay (Molecular Probes, Eugene, Oregon, USA) as described by the manufacturer. Briefly, cell culture medium was replaced with 400 µL of PBS-calcein AM (1 µg/mL). Cells were them incubated 15 minutes at 37°C. Fluorescent images were obtained in an Olympus BX51 microscope.

#### 7.2. Cellular morphology study

Cell cultured scaffolds were fixed with Formaldehyde 3.7%. For cellular observation with SEM (JEOL JSM-35 CF), some samples were fixed with Formaldehyde 3.7%, dehydrated in an alcohol gradient and prepared by gold-coating using a sputter coater (Pelco 91000). For confocal microscopy, other samples were also fixed with Formaldehyde 3.7%. In these samples F-actin was labelled with Texas Red-Phalloidin (Molecular Probes, Eugene, Oregon, USA) and nucleus was stained with Hoechst (Molecular Probes, Eugene, Oregon, USA) as described by the manufacturer. Briefly, Formaldehyde solution was removed and samples were washed twice with PBS. Then they were incubated with 0.1% Triton X-100 in PBS during 5 minutes and washed again with PBS. Finally samples were incubated with the inmunostaining solution 30 minutes and washed twice with PBS. Images were obtained and analyzed using Leica Lite software.

#### 7.3. Magnetic resonance imaging (MRI)

Both control and cell-seeded scaffolds were transferred to 1.5 mL tubes and visualized by MRI. Data was acquired using a 4.7 Tesla Bruker BIOSPEC 47/40 MRI system with a gradient intensity of 45 G/cm. A Bruker designed volume coil was used for data acquisition (diameter = 3.5 cm). 2D proton-weighted fast spin echo (FSE) MR images were acquired with the following settings: repetition time (TR) = 3000 ms; Echo time (TE) = 20 ms; slice thickness = 1.5 mm; field of view (FOV) = 1×1 cm^2^; matrix = 256×256. The resulting resolution was 39 µm×39 µm. 3D proton-weighted FSE-MR images were also acquired with the following settings: TR = 3000 ms; TE = 20 ms; FOV = 1×1×1 cm^2^; matrix = 256×192×192. These data was reconstructed to yield a reconstructed matrix size of 256×256×256, with a resolution of 39 µm×56 µm×56 µm.

#### 7.4. MTS cell proliferation assay

It was performed as described by the manufacturer (Aqueous MTS Non-Radioactive Cell Proliferation Assay, Promega, Madison, WI, USA). Briefly, scaffolds were transferred to new wells, reconstituted MTS was added (40 µl MTS in 400 µl medium) and scaffolds were incubated at 37°C for 90 min. The medium was transferred to new wells to measure the absorbance (460 nm, Biotek FL-600) and blank readouts were subtracted. The data obtained were converted to cell number by interpolation on a standard curve.

#### 7.5. Alamar Blue cell proliferation assay

It was performed as described by the manufacturer (Biosource, Camarillo, CA, USA). Briefly, before each measurement scaffolds were transferred to new wells and new culture medium and Alamar Blue reagent were added (40 µl of reagent in 400 µl of medium). After the incubation period (37°C, 120 min.) the medium was transferred to new wells, the absorbance (590 nm) was measured (Biotek FL-600) and blank readouts were subtracted. The data obtained were converted to cell number by interpolation on standard curve.

#### 7.6 In vitro testing of BMP-2 activity

The effect of the scaffold-adsorbed protein was evaluated through the colorimetric measurement of alkaline phosphatase (ALP) activity. Briefly described, after the removal of culture medium, scaffolds were washed with PBS (200 µL). Afterwards, a 100 µL/well of lysis buffer (50 mM Tris pH 6.8, 0.1% Triton X-100, 2 mM MgCl_2_) was added. 10 µL samples were assayed for alkaline phosphatase activity in 96-well plates, using p-nitrophenylphosphate in 2-amino-2-methyl-1-propanol buffer as a substrate in a total volume of 100 µL; after 10 min. at 37°C. The reaction was stopped with 100 µL of 0.5 M NaOH and the absorbance was measured at 450 nm on a Microplate Reader (Biotek FL-600).

### 8. Experimental in animals (rabbit models)

The *in vivo* studies were performed in New Zealand male rabbits, which had an average weight of 3 kg. The rabbits were anesthetized by intramuscular injection of 2% Rompun (Xylacine 1 mL/10 kg, Bayer) and Imalgene 1000 (ketamine 20 mg/kg, Merial). Then, the surgical area was shaved and washed with an antiseptic solution (Betadine, 10% povidone-iodine, Meda Manufacturing, France). For muscle implantation, an incision was made in the dorsal muscle tissue, samples were inserted (control samples (n = 6) and BMP-2 carrier samples (n = 6)). For bone implantation, an incision was made in the periostium of the tibial plateau. Later a defect was drilled and a sample was inserted in the defect (control samples in the right legs (n = 5) and BMP-2 carrier samples in the left legs (n = 5)). In all cases wound was sutured layer-by-layer. Animals were sacrificed after three weeks and samples were dissected and fixed in formalin solution for subsequent studies.

### 9. Experimental in animals (pig model)

Pigs (n = 8) were anesthetized with a mixture of Medetomidin (0.03 mg/kg of Domtor®, Pfizer, Dublin, Ireland), ketamine (10 mg/kg of Ketolar®, Pfizer) and atropin sulfate (0.02 mg/kg Atropina Braun®, Braun Surgical SA, Rubí, Barcelona, Spain) delivered by intramuscular injection. During the surgery the animals were monitorized and anaesthesia was maintained with Propofol 1%. 2 defects were created in each pig. In each case, the gingiva was open and palatal flap was elevated. From distal to palatal, and below the root of the teeth, a critical size defect was created (1.5 cm×1 cm×1 cm) and a scaffold was inserted in the defect. The SFF implants (1.5 cm×0.9 cm×0.9 cm) were fixed with 2 screws. Bio-Oss® samples were cut to fit to defect area and fixed with surgery glue (histoacryl® surgical glue, Braun Surgical, Tuttlingen, Germany). After surgery, daily during seven days, the animals received Amoxicilin (7 mg/10 kg) and clavulamic acid (1.75 mg/19 kg of Syinulox®, Pfizer) by intramuscular injection. Pigs were euthanized 3 months after surgery by intravenous injection of 100 mg/kg of sodium pentobarbital. Samples were harvested and screws were removed for μCT and histological evaluation.

#### 9.1. 3D imaging and sample measurements

Before the histological analysis 13 from all 16 samples were observed by microcomputed tomography (μ-CT) as described above (see 4.3.). All measurements and 3D surface reconstructions were performed with MicroView ABA 2.2 software (GE Healthcare). Mean and standard deviation were obtained from “Bone Volume” and “Bone Mineral Content” parameters. Statistical analysis was performed using GraphPad Prism version 5.00 for Windows, (GraphPad Software, San Diego, California, USA). One-way ANOVA Analysis with Dunnet post-test between all treatments and control (empty defect) was performed. Significative differences stands for: * (p≤0.05), ** (p≤0.01), *** (p≤0.001).

#### 9.2. Histology

Samples were decalcified with 10% nitric acid during 3 days. After dehydration, decalcified samples were paraffin-embedded and longitudinally sectioned for histological study. Three different serial slices were obtained in each sample (10 slices in each series). Hematoxylin and eosin and Masson's tricrome stainings were performed. The histological processing was performed by Dominion-Pharmakine histology services (www.pharmakine.com). Stained slides were viewed with an Olympus BX51 microscope.

## Supporting Information

Movie S1 **3D MRI study.** Video shows the 3D reconstruction of an entire scaffold. Brightness corresponds to seeded cells which colonize all structure.
(MOV)
